# CDK2 phosphorylation of Werner protein (WRN) contributes to WRN’s DNA double‐strand break repair pathway choice

**DOI:** 10.1111/acel.13484

**Published:** 2021-10-06

**Authors:** Jong‐Hyuk Lee, Raghavendra A. Shamanna, Tomasz Kulikowicz, Nima Borhan Fakouri, Edward W. Kim, Louise S. Christiansen, Deborah L. Croteau, Vilhelm A. Bohr

**Affiliations:** ^1^ Section on DNA Repair National Institute on Aging National Institutes of Health Baltimore MD USA; ^2^ Danish Center for Healthy Aging University of Copenhagen Copenhagen Denmark; ^3^ Present address: Benevir The Janssen Pharmaceuticals Companies of Johnson & Johnson Rockville Maryland 20850 USA

**Keywords:** aging, DNA double strand break, DNA repair, phosphorylation, Werner Syndrome

## Abstract

Werner syndrome (WS) is an accelerated aging disorder characterized by genomic instability, which is caused by WRN protein deficiency. WRN participates in DNA metabolism including DNA repair. In a previous report, we showed that WRN protein is recruited to laser‐induced DNA double‐strand break (DSB) sites during various stages of the cell cycle with similar intensities, supporting that WRN participates in both non‐homologous end joining (NHEJ) and homologous recombination (HR). Here, we demonstrate that the phosphorylation of WRN by CDK2 on serine residue 426 is critical for WRN to make its DSB repair pathway choice between NHEJ and HR. Cells expressing WRN engineered to mimic the unphosphorylated or phosphorylation state at serine 426 showed abnormal DSB recruitment, altered RPA interaction, strand annealing, and DSB repair activities. The CDK2 phosphorylation on serine 426 stabilizes WRN’s affinity for RPA, likely increasing its long‐range resection at the end of DNA strands, which is a crucial step for HR. Collectively, the data shown here demonstrate that a CDK2‐dependent phosphorylation of WRN regulates DSB repair pathway choice and cell cycle participation.

## Graphical abstract

When DSBs occurs in the G1 phase of the cell cycle, the KU70/80 complex interacts directly with WRN and stimulates WRN's exonuclease activity. Stimulated by KU70/80, DNA‐PKcs also regulates WRN's enzymatic activities. Finally, fully activated WRN utilizes its nuclease activity to create DNA ends suitable for XRCC4‐DNA ligase IV complex mediated ligation. When cells progress into S phase of the cell cycle, CDK2 becomes active via CycE, and phosphorylates WRN on S426. S426 phosphorylated WRN has increased affinity for RPA, thereby promoting end resection and stabilization of the resected single‐stranded DNA. Also, S426 phosphorylation enhances WRN's strand annealing activity to promote strand annealing activity to form D‐loop formation for efficient HR.

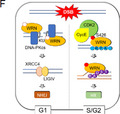

## 1 INTRODUCTION

Werner syndrome (WS) is a rare autosomal recessive genetic disorder caused by mutation in the *WRN* gene (Bohr, 2005). This premature aging disorder is characterized by scheduled hierarchical deterioration of connective tissue and of the endocrine‐metabolic system, as also seen in other diseases of accelerated aging (Oshima et al., 2017). WS patients suffer from cancer, diabetes, cardiovascular disease, and die at a median age of 54 (Huang et al., 2006). WS patients also have much higher incidence of sarcomas than age‐matched normal individuals (Goto et al., 1996; Lauper et al., 2013), suggesting that WRN plays a central role in maintaining genome stability.

WRN is a member of the RecQ helicase family of proteins and has strand annealing and exonuclease activities (Croteau et al., 2014). It is rapidly recruited to the site of DNA damage and interacts with a number of DNA repair proteins, participating in base excision DNA repair (BER), classical/alternative non‐homologous end joining (NHEJ), homologous recombination (HR), and replication re‐start after DNA damage (Chen et al., 2003; Croteau et al., 2014; Lachapelle et al., 2011; Oshima et al., 2002). WS patients and cells lacking WRN show significantly increased sensitivity to DNA damaging agents, highlighting WRN’s key role in DNA repair (Shamanna et al., 2016). Recently, it was shown that WRN regulates the DNA double‐strand break (DSB) repair pathway choice between classical NHEJ (c‐NHEJ) and alternative NHEJ (alt‐NHEJ)(Shamanna et al., 2016). WRN promotes Ku‐dependent c‐NHEJ with its catalytic activities and strongly inhibits alt‐NHEJ with non‐enzymatic activities, while downregulating the recruitment and downstream functions of MRE11 and CtIP to inhibit alt‐NHEJ.

The DSBs are among the most harmful types of cellular DNA damage, causing devastating effects including mutagenic changes, developmental defects, gross chromosomal rearrangements, cell death, and malignancy (Huertas & Jackson, 2009). In higher eukaryotes, DSBs are mostly repaired by HR, single‐strand annealing (SSA), and NHEJ (Ceccaldi et al., 2016). The choice of DNA repair pathway utilized is tightly regulated by multiple molecular mechanisms and is closely related to cell cycle progression. NHEJ is active throughout the cell cycle but also error prone, as the process does not use a complementary template to guide DNA repair and instead directly ligates the broken DNA ends (Ceccaldi et al., 2016). HR, on the other hand, is an error‐free repair mechanism which uses a replicated sister chromatid to guide the DSB repair. DSBs that occur in S and G2 cell cycle phases are preferentially repaired by HR (Johnson & Jasin, 2000), otherwise, NHEJ dominates.

WRN interacts with multiple proteins in the NHEJ pathway such as KU70/80 (Cooper et al., 2000), DNA‐PKcs (Yannone et al., 2001), XRCC4, and DNA ligase IV (Kusumoto et al., 2008). KU70/80 forms a stable complex with DNA‐PKcs on DNA damage sites and initiates the NHEJ signaling pathway (Walker et al., 2001). The KU70/80 complex interacts directly with WRN and strongly stimulates WRN’s exonuclease activity (Cooper et al., 2000; Li & Comai, 2000). Stimulated by KU70/80, DNA‐PKcs also phosphorylates and regulates WRN’s enzymatic activities (Karmakar et al., 2002; Yannone et al., 2001). Finally, WRN utilizes its nuclease activity to create DNA ends suitable for XRCC4‐DNA ligase IV complex‐mediated ligation (Kusumoto et al., 2008).

The key to accurate DSB repair is the precise regulation of the processing of DNA ends. Resection of DNA is relatively limited during c‐NHEJ, while quite extensive during HR and alt‐NHEJ (Howard et al., 2020; Ira et al., 2004). During HR and alt‐NHEJ, the initial resection (short range) is regulated by the MRN complex and CtIP whereas the extended resection (long‐range) is regulated by DNA2/BLM or EXO1(Cejka, 2015). Replication Protein A (RPA) is one of WRN’s strongest interacting partners and greatly stimulates its DNA unwinding activity (Brosh et al., 1999; Shen et al., 1998). WRN is stimulated to unwind DNA by RPA, generating longer single‐stranded DNA for a proper recombination reaction (Brosh et al., 1999; Lee et al., 2018). Since WRN is recruited to the site of laser‐induced DSB sites in G1 as well as in S and G2 cell cycle phases (Shamanna et al., 2016), this evidence suggests that it can participate in both NHEJ and HR. However, the molecular mechanism behind the pathway choice between NHEJ and HR is not yet understood.

Here, we performed laser‐induced DSB recruitment assays in U2OS cells to investigate the upstream regulation of WRN by CDK2 and found that CDK2 inhibition significantly altered the recruitment dynamics of WRN to the DSB. *In vitro* assays confirmed the direct phosphorylation of WRN on its serine 426 residue by CDK2. To delineate the significance of the phosphorylation on this residue, NHEJ and HR assays were performed in cells expressing WRN S426 residue point mutants. Cells expressing a phosphomimetic WRN showed increased binding affinity to RPA and recovered NHEJ and HR efficiency similar to that of the wild type. In contrast, cells expressing S426 non‐phosphorylatable WRN showed decreased strand annealing, increased NHEJ, and decreased HR activity. Altogether, these results suggest that CDK2 regulates WRN’s pathway choice between NHEJ and HR, and that phosphorylation of WRN by CDK2 increases its binding affinity to RPA thereby possibly stabilizing resected single‐stranded DNA.

## 2 RESULTS

**FIGURE 1:**
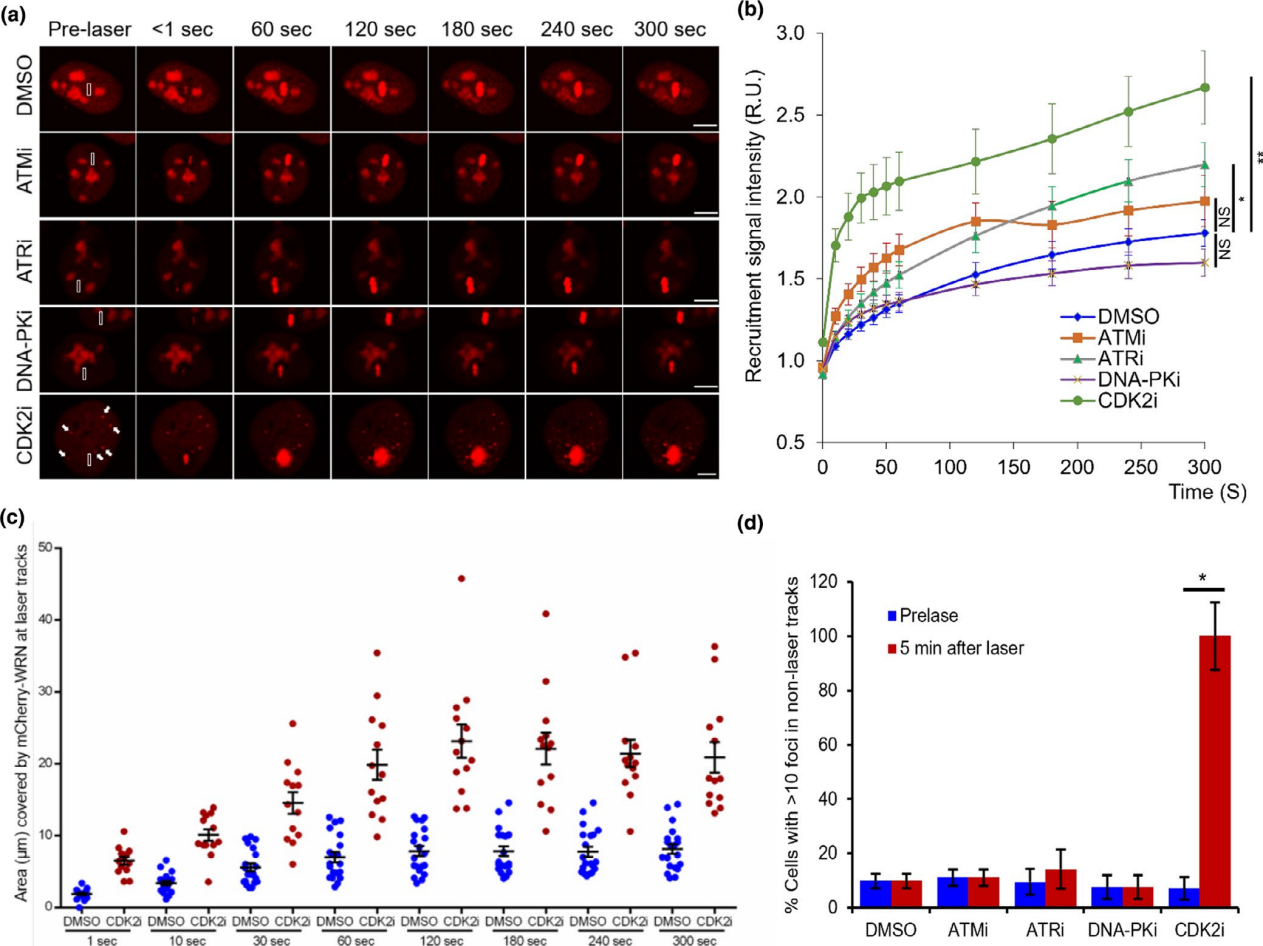
Inhibition of CDK2 activity enhances WRN recruitment to DSBs. (a) Recruitment of mCherry‐WRN to laser tracks. U2OS cells expressing mCherry‐WRN were treated with DMSO, 100 μM ATMi for 2 h, 100 μM ATRi for 3 h, 100 μM DNA‐PKi for 2 h and CDK2i (25 μM) for 4 h, and micro‐irradiated with 435 nm laser to induce DSBs in 3 μm × 0.25 μm tracks (white boxed area). Images were captured at 10 sec intervals for 5 min. (b) Real‐time recruitment of mCherry‐WRN to DSB tracks in cells treated with inhibitors as in panel A. Scale bar, 5 μm. (c) Graph showing area occupied by mCherry‐WRN at laser‐induced DSB tracks. Blue, DMSO; Red, CDK2i. (d) Graph showing quantitation of cells with >10 mCherry‐WRN foci in non‐laser tracks, which are indicated in (a) (white arrows). n = 15 (DMSO), n = 15 (ATMi), n = 19 (ATRi), n = 13 (DNA‐PKi), n = 12 (CDK2i) cells. Error bars represent standard deviation. *p*‐value, *, <0.05; **, <0.01

The recruitment and activity of WRN are regulated by many proteins. ATR regulates WRN’s subnuclear re‐localization and interaction with RPA to prevent DSB formation at stalled replication forks (Ammazzalorso et al., 2010; Patro et al., 2011). ATM‐dependent phosphorylation of WRN is important for the recovery of collapsed forks and allows RAD51 accumulation in foci (Ammazzalorso et al., 2010). DNA‐PKcs, which forms a complex with KU at broken DNA ends, also regulates WRN’s exonuclease and helicase activities (Karmakar et al., 2002; Kusumoto‐Matsuo et al., 2010). CDK2 is a cell cycle regulator that initiates entry into S phase, a signal that activates DNA replication (Caruso et al., 2018). WRN is recruited to the DSB and plays important roles in multiple steps during NHEJ and HR (Lu & Davis, 2021), which is a dominant DSB repair especially during S and G2 phase (Johnson & Jasin, 2000). To test if CDK2 regulates WRN’s DSB recruitment efficiency, mCherry‐WRN expressing U2OS cells were treated with CDK2‐specific inhibitor (CDK2i) and we then compared the influence of CDK2i with that of ATM, ATR, and DNA‐PKcs activities on WRN’s recruitment dynamics. Of the inhibitors tested, only CDK2i altered WRN’s subcellular distribution, which otherwise is mainly localized in the nucleoli (Figure 1a, CDK2i panel). Live‐cell confocal imaging indicated that mCherry‐WRN robustly recruited to DSB tracks in DMSO‐treated cells (Figure 1a,b). WRN’s recruitment kinetics was investigated in the presence of DNA‐PKcs‐, ATM‐, ATR‐, or CDK2‐specific inhibitors (Figure 1a,b). Notably, inhibiting CDK2 significantly increased the signal intensity (1.5‐fold increase, endpoint) and enlarged the area of WRN recruitment at the laser‐induced DSB loci (Figure 1b,c). Moreover, CDK2 inhibition induced WRN foci formation outside the laser tracks (Figure 1a, arrows and d). Together, these results suggest that CDK2 regulates WRN’s foci forming activity.

**FIGURE 2:**
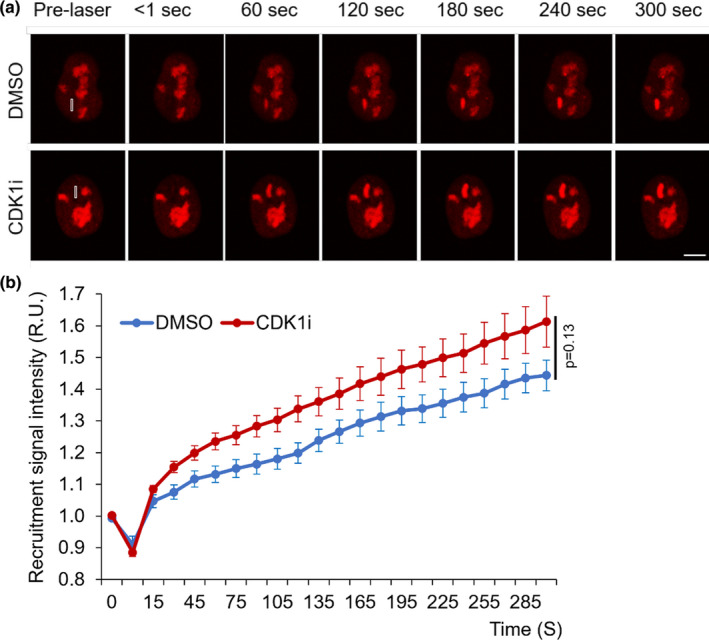
Inhibition of CDK1 activity marginally increases mCherry‐WRN recruitment to DSBs. (a) Recruitment of mCherry‐WRN to laser tracks. U2OS cells expressing mCherry‐WRN were treated with CDK1i (25 μM) for 4 h, and micro‐irradiated with 435 nm laser to induce DSBs in 3 μm × 0.25 μm tracks. Images were captured at 10 sec intervals for 5 min. Scale bar, 5 μm. (b) Real‐time recruitment of mCherry‐WRN to DSB tracks in cells treated with CDK1i as in panel A. n = 19 (DMSO), n = 21 (CDK1i) cells

It is generally considered that D‐type cyclins and CDK4 or CDK6 regulate events in early G1 phase, CDK2‐CycE triggers entry into S phase, CDK2‐CycA and CDK1‐CycA regulate the completion of S phase, and CDK1‐CycB is responsible for mitosis (Hochegger et al., 2008; Hume et al., 2020). A recent study using Roscovitine, a CDK2, CDK7, and CDK9 inhibitor in cells, identified that CDK1 phosphorylates WRN at Serine‐1133 on collapsed replication forks (Palermo et al., 2016). To compare the effect of CDK1 on WRN’s recruitment to DSBs, we transfected U2OS cells with mCherry‐WRN expressing plasmids and treated with CDK1‐specific inhibitor. Real‐time imaging of mCherry‐WRN’s recruitment indicated that inhibition of CDK1 activity minimally (~10%) affected the recruitment of WRN to DSBs (Figure 2). Unlike CDK2i (Figure 1a and d), CDK1 inhibition did not induce WRN foci formation outside the laser tracks (Figure 2a) and did not drastically alter the distribution of WRN at DSB tracks (compare Figure 1 with Figure 2, 1.5‐ vs ~1.1‐fold change at the endpoint).

**FIGURE 3:**
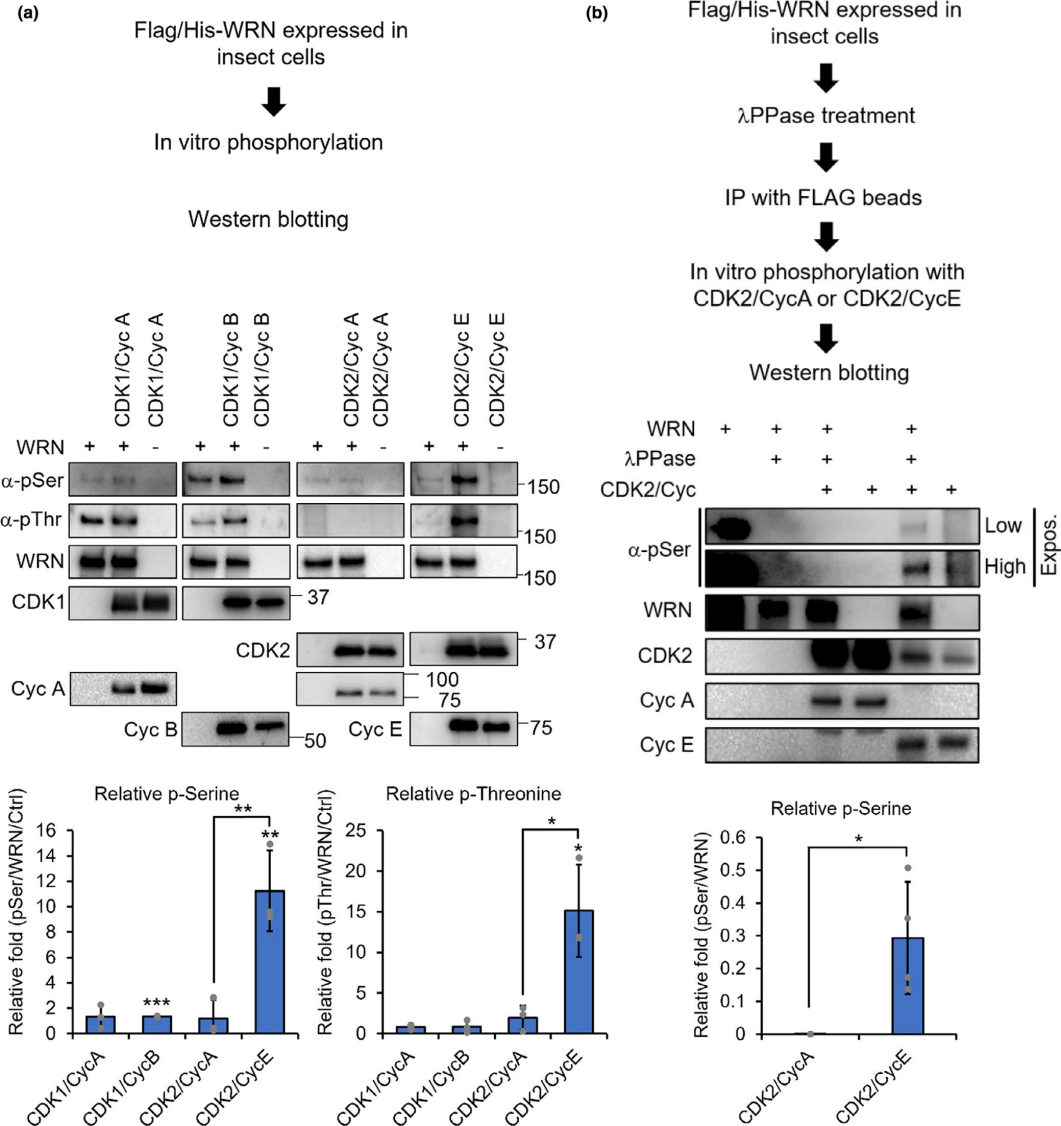
CDK2‐CycE phosphorylates WRN. (a) FLAG/His‐tagged WRN protein was purified from baculovirus‐infected insect cells. The purified WRN protein was *in vitro* phosphorylated with the indicated CDK‐Cyc combinations and probed by Western blot for the antigens shown. Phosphorylated serine/threonine blot intensities were quantified by normalizing with WRN and basal phosphorylation without CDKs. Asterisks indicated directly above the columns represent p‐value calculations compared to controls (basal phosphorylation without CDKs). (b) To remove the basal phosphorylation seen in A, purified WRN proteins were treated with λPPase, then immunoprecipitated with FLAG beads and subjected to *in vitro* phosphorylation with CDK2‐CycA or CDK2‐Cyc2 combinations, followed by Western blot analysis. Phosphorylated serine blot intensity was quantified by normalizing with WRN. Error bars represent standard deviation. *p*‐value, *, <0.05; **, <0.01; ***, <0.001

As CDKs are a family of kinases that transduce downstream signaling via phosphorylation, we tested whether they could phosphorylate WRN directly. Purified FLAG/His‐tagged WRN proteins were subjected to *in vitro* phosphorylation with different CDK‐cyclin combinations. When purified from the insect cells, high levels of basal WRN phosphorylation were observed (Figure 3a, first lane of each panel). CDK1‐CycA showed no effect and CDK1‐CycB had small, but highly significant effect (~1.3‐fold, compared to basal phosphorylation level) on WRN phosphorylation (Figure 3a). While CDK2‐CycA had no effect on WRN (Figure 3a, third panel), the CDK2‐CycE combination phosphorylated WRN more than ~10‐fold, compared to control (Figure 3a, fourth panel, phosphorylated serine/threonine levels of WRN by different combinations of CDK/Cyc combinations are shown in blot quantification). To eliminate the basal phosphorylation and identify the role of CDKs in phosphorylating WRN, purified proteins were treated with λPPase, immunoprecipitated with FLAG conjugated beads, and tested for *in vitro* phosphorylation. Consistent with the laser‐induced WRN recruitment experiments (Figures 1 and 2), CDK2‐CycE significantly phosphorylated WRN while CDK2‐CycA had no effect (Figure 3b, fifth lane compared to the third lane, the blot quantification), indicating that WRN is a substrate for the CDK2‐CycE complex.

**FIGURE 4:**
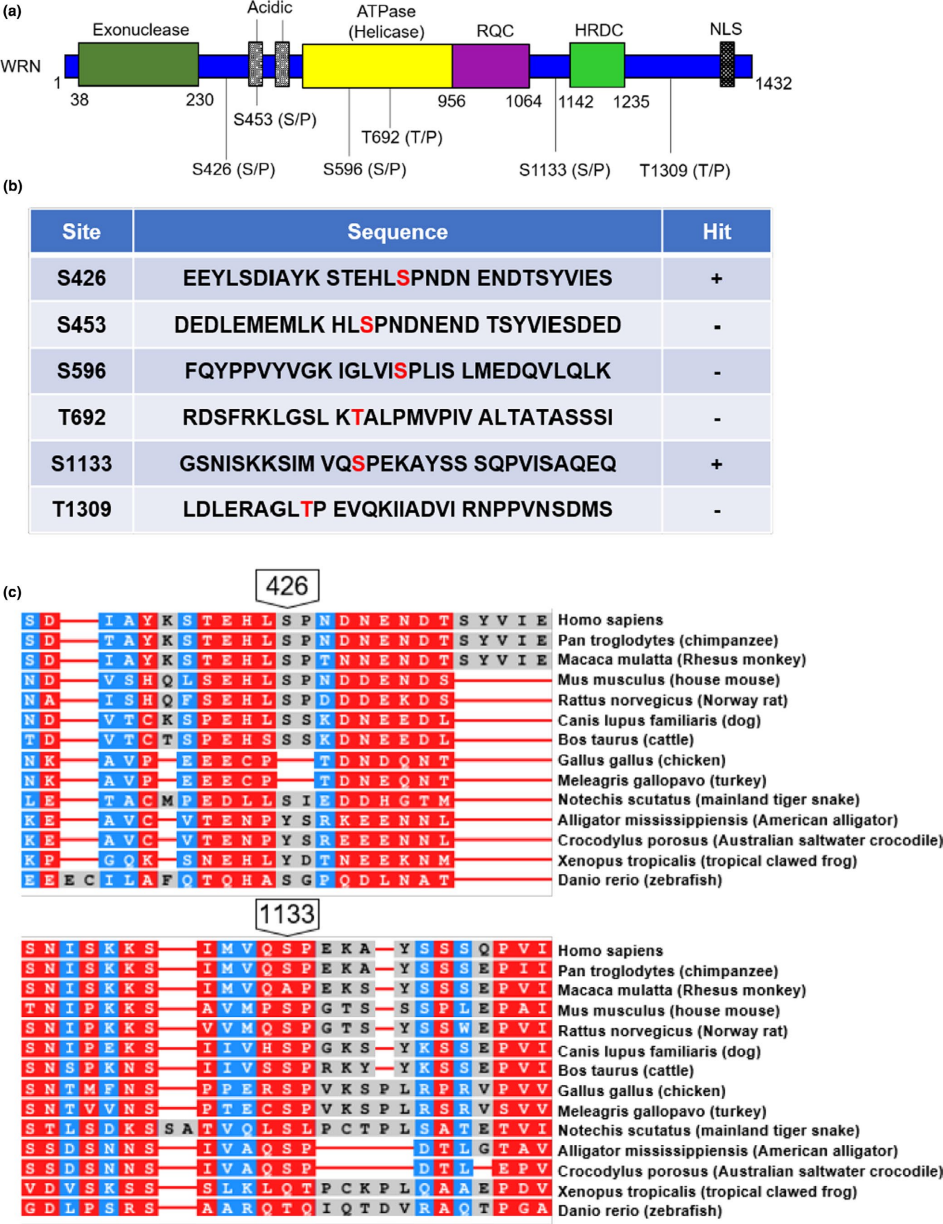
Identification of CDK2 phosphorylation site on WRN. (a) Potential phosphorylation sites for CDK2 on WRN. Schematic domain structure and their amino acid numbers are depicted. (b) FLAG/His‐tagged WRN proteins were purified from baculovirus‐infected insect cells. Purified WRN proteins were then treated with λPPase to eliminate basal phosphorylation and subjected to CDK2‐CycE *in vitro* phosphorylation. Phosphorylation products were separated by SDS‐PAGE, and WRN protein bands were analyzed with mass spectrometric analysis. (c) Amino acid sequence conservation analysis using Constraint‐based Multiple Alignment Tool (NCBI). Red indicates highly conserved positions and blue indicates lower conservation

To identify the site(s) of phosphorylation on WRN, purified and λPPase‐treated WRN proteins were *in vitro* phosphorylated with the CDK2‐CycE complex and subsequently resolved on SDS‐PAGE (Supplementary Figure S1), after which WRN‐specific bands were excised and analyzed by mass spectrometry. Among the 6 candidate CDK2 phosphorylation substrate sites (Figure 4a), serine residues 426 and 1133 showed positive hits (Figure 4b, full list can be found on Supplementary Table S2). We found that the amino acid sequences of these two residues are conserved among species (Figure 4c). S426 seemed more conserved among higher eukaryotes, especially in mammals, while S1133 showed broader conservation among species tested. Since S426 is less explored, we focused on this site testing the effect of phosphorylation.

**FIGURE 5:**
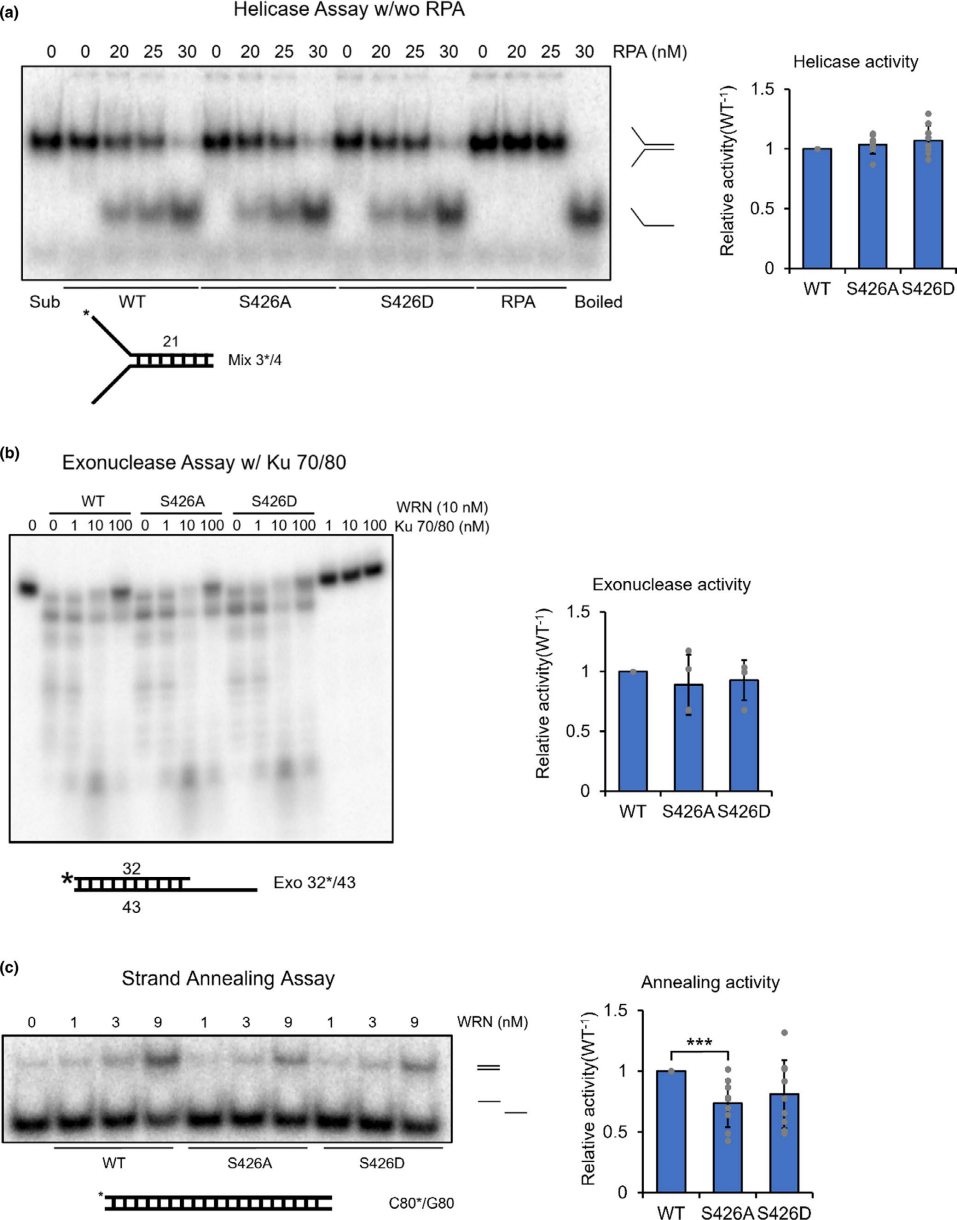
Phosphorylation deficient WRN has decreased strand annealing activity. (a) WRN’s *in vitro* helicase activity comparison. Wild‐type and point mutant WRN proteins in combination with RPA proteins were incubated with radiolabeled 21‐bp forked dsDNA substrate. (b) WRN’s *in vitro* exonuclease activity comparison. WRN proteins were incubated with Ku 70/80 heterodimer to test their basal and stimulated exonuclease activity on 5’‐overhang DNA substrate. (c) Indicated concentrations of wild‐type and point mutant WRN proteins were incubated with radiolabeled C80 and G80 ssDNA oligonucleotides. Blot quantification data shown (relative annealing activity was calculated by normalizing the blot intensity with WT in each concentration group. n = 3). Error bars represent standard deviation. *p*‐value, ***, <0.001. Reaction products were separated using native PAGE and analyzed by phosphorimaging

To test the effect of the S426 phosphorylation, we generated single amino acid substitution mutants of WRN. We then performed a set of biochemical experiments to understand if S426 phosphorylation of WRN affects its enzymatic activity in DNA metabolism. *In vitro* helicase assays confirmed that S426A, phosphorylation‐deficient alanine substitution mutant, or S426D, phosphomimetic aspartate substitution mutant, of WRN did not affect WRN’s double‐stranded DNA unwinding activity in the absence or presence of RPA, relative to WT WRN’s activity (Figure 5a). Also, S426 phosphorylation status did not affect the exonuclease activity of WRN itself nor in cooperation with KU70/80 (Figure 5b). Interestingly, the S426A WRN showed significantly decreased strand annealing activity (~30%), compared to wild‐type WRN (Figure 5c).

In order to test the effect of S426 phosphorylation *in vivo*, we tried to generate stable cell lines expressing WT, S426A, or S426D mutant WRN. However, knockdown of endogenous WRN in U2OS cells resulted in severe growth arrest and a senescent phenotype, making it unfeasible to generate stable cell lines (data not shown). This agrees with previous reports claiming that downregulation of WRN leads to disrupted redox homeostasis and proliferation impairment in cancer cells (Li et al., 2014).

**FIGURE 6:**
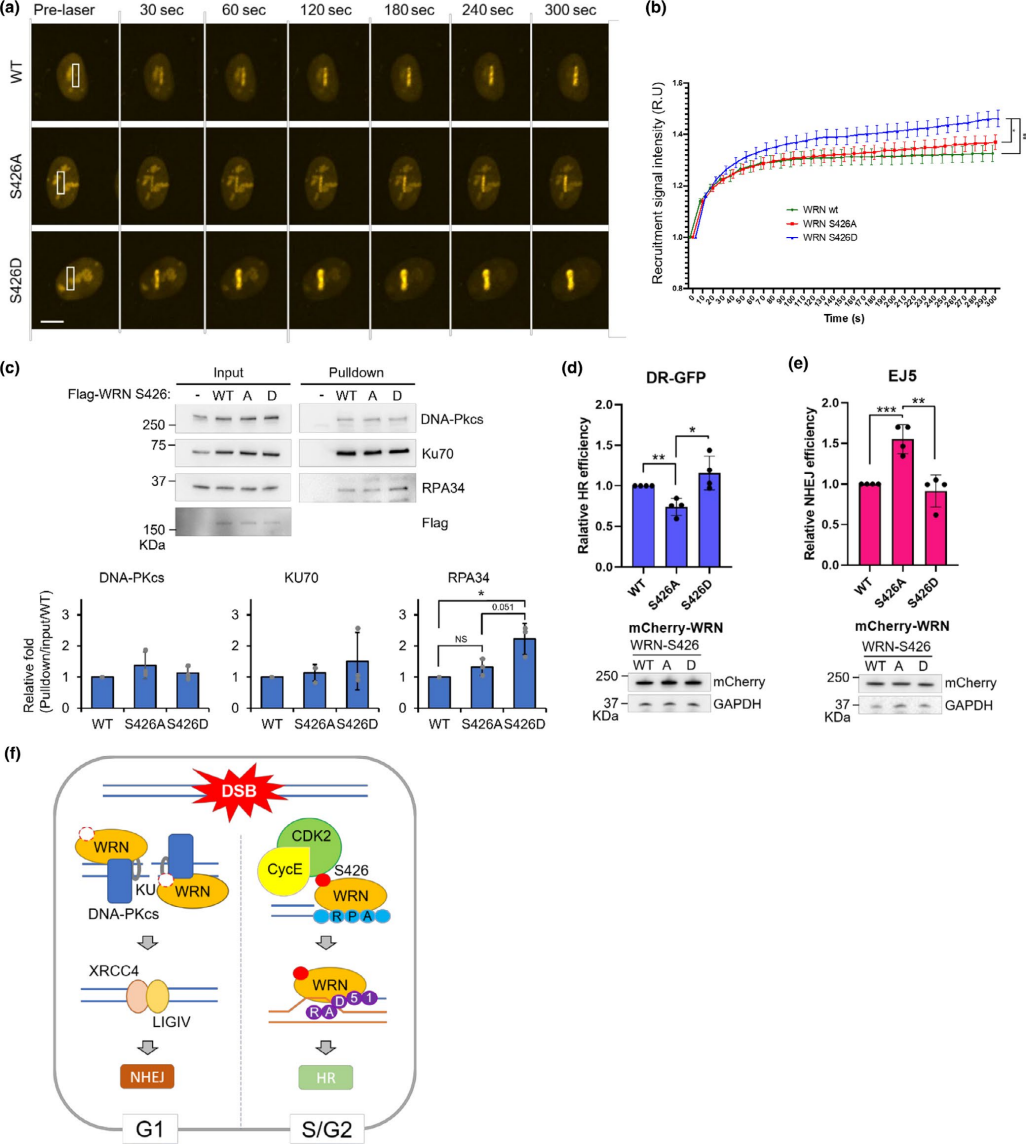
Phosphorylation of S426 by CDK2 shifts WRN’s DNA repair activity toward homologous recombination. (a) Recruitment of mCherry‐WRN to laser tracks. U2OS cells expressing mCherry‐tagged WT/S426A/D‐WRN were micro‐irradiated with 435 nm laser to induce DSBs. Images were captured at 10 sec intervals for 5 min. Scale bar, 10 μm. n = 3 for each group. (b) Real‐time recruitment of mCherry‐WRN to DSB tracks in cells as in panel A. (c) U2OS cells were γ‐irradiated (10 Gy), 30 min after irradiation, cells were lysed and mixed with FLAG‐tagged purified WT/S426A/D‐WRN protein for binding assay. Indicated WRN interacting proteins were probed by Western blot for the antigens shown. (d) HR efficiency in DR‐GFP cells. (e) Total NHEJ efficiency in EJ5 cells. DR‐GFP and EJ5 cells were induced by co‐transfecting I‐SceI and mCherry WT/S426A/D‐WRN expression plasmid. Graph represents relative repair efficiency as measured by GFP positive cells normalized to mCherry expression. Immunoblots represent protein expression levels. (f) Schematic diagram of CDK2‐mediated WRN phosphorylation on DSB pathway choice. When DSBs occur in the G1 phase of the cell cycle, the KU70/80 complex interacts directly with WRN and stimulates WRN’s exonuclease activity. Stimulated by KU70/80, DNA‐PKcs also regulate WRN’s enzymatic activities. Finally, fully activated WRN utilizes its nuclease activity to create DNA ends suitable for XRCC4‐DNA ligase IV complex‐mediated ligation. When cells progress into S phase of the cell cycle, CDK2 becomes active via CycE, and phosphorylates WRN on S426. S426 phosphorylated WRN has increased affinity for RPA, thereby promoting end resection and stabilization of the resected single‐stranded DNA. Also, S426 phosphorylation enhances WRN’s strand annealing activity to promote strand annealing activity to form D‐loop formation for efficient HR. Error bars represent SD from four independent experiments. *p*‐value, *, <0.05; **, <0.01; ***, <0.001

The mutants were tagged with mCherry and introduced to U2OS cells for visualization in laser‐induced DSB recruitment experiments. S426D mutation showed significantly increased recruitment affinity to the laser‐induced DSB, compared to the wild‐type and S426A mutant WRN (Figure 6a,b). Theoretically, the S426A mutant was expected to mimic the CDK2 inhibition when WRN protein is unphosphorylated. However, S426A WRN’s recruitment to the DSB showed somewhat opposite results from the CDK2 inhibition experiment (Figure 1a, b). Notably, it did not show robust accumulation. We should note that in Figure 1a, WRN’s nucleolar subcellular distribution is perturbed relative to all other pre‐laser images. Thus, it is likely that CDK2 inhibition induces complex biological effects which cannot be replicated by a single amino acid substitution. Despite these conflicts, these data clearly suggest that the phosphorylation of S426 residue modulates WRN’s recruitment to DSBs.

To understand the implications of WRN’s S426 phosphorylation, we investigated protein interactions between DNA repair factors and the WRN mutants. Wild‐type and mutant variations of FLAG‐tagged WRN proteins were purified and incubated with gamma‐irradiated U2OS cell lysates, after which interacting proteins were evaluated by pull‐down experiments. The KU70/80 heterodimer interacts with WRN and closely associates with DNA‐PKcs to initiate a cascade of events that constitutes the NHEJ pathway (Walker et al., 2001). RPA is one of the strongest interacting partners of WRN and important for stabilization of single‐stranded DNA for higher efficiency recombination during HR (Brosh et al., 1999; Lee et al., 2018; Shen et al., 1998). WRN’s interaction affinity with DNA‐PKcs and KU70 was not altered by S426A or D mutations. Surprisingly, WRN’s interaction with RPA was significantly increased by the S426D mutation (Figure 6c). We then tested if RPA recruitment to the laser‐induced DSBs would be affected by the WRN S426 mutation. However, there were minimal differences in RPA recruitment to the DSBs with WRN S426A or D mutation (Supplementary Figure S2). Consistent with the notion that RPA recruitment occurs prior to that of WRN at DSBs.

To understand the physiological relevance of S426 phosphorylation of WRN on DNA repair, we performed HR and NHEJ DNA repair assays using DR‐GFP and EJ5 reporter systems (Gunn et al., 2011; Mao et al., 2008; Seluanov et al., 2004). WRN’s S426A mutation significantly reduced the efficiency of HR, while the S426D mutation functioned like WT (Figure 6d). On the contrary, the S426A mutation drastically increased NHEJ efficiency while the S426D mutation abolished this effect (Figure 6e). Taken together, these data suggest that phosphorylation of WRN on the S426 residue is critical for the pathway choice decision between HR and NHEJ.

## 3 DISCUSSION

In our previous work, we observed WRN recruitment to DSBs during all phases of the cell cycle (Shamanna et al., 2016), showing that WRN could participate in both the HR and the NHEJ pathways during DSB repair. In addition to its key role in NHEJ (Shamanna et al., 2016), WRN is also suggested to play a role in HR (Palermo et al., 2016; Shamanna et al., 2017). In this study, we discovered that inhibiting CDK2 activity significantly affected WRN’s DSB recruitment dynamics. In order to investigate this altered WRN dynamics in more detail, we performed chromatin immunoprecipitation (ChIP) using WRN antibody. We tested many antibodies including commercially available ones as well as our own homemade, targeting diverse epitopes on WRN protein. Unfortunately, none of the existing antibodies were suitable for ChIP application (data not shown). This may reflect WRN’s complex and dynamic interaction with chromatin and other DNA metabolism‐related factors.

We found that CDK2‐CycE phosphorylates serine 426 of WRN. CDKs phosphorylate many DNA repair proteins including NBS1, CtIP, EXO1, DNA2 (Chen et al., 2011; Falck et al., 2012; Huertas & Jackson, 2009; Tomimatsu et al., 2014; Wang et al., 2013), and another RecQ family helicase RECQL4 to facilitate the resection process (Lu et al., 2017). During the S/G2 phase, CDK1 and 2 phosphorylate RECQL4, inducing DSB recruitment by enhancing its binding affinity to MRE11. It has been shown that phosphorylation of RECQL4 stimulates its helicase activity, promotes DNA end resection to increase HR and cell survival after ionizing radiation, and prevents cellular senescence. Interestingly, primary fibroblast cells showed similarly increased persistent DNA damage after WRN or RECQL4 knockdown, suggesting that CDK regulation of WRN and RECQL4 have functional similarities in the DSB response.

At the end of G1 phase of mammalian cells, the CDK2‐CycE complex reaches maximum activity and initiates S phase entry (Classon & Harlow, 2002). As cells progress through S phase, CycE is degraded through ubiquitin‐mediated proteasomal degradation and CDK2 activity is maintained by cyclin A throughout S and G2 phase (Koepp et al., 2001). In our data, we showed that WRN is highly phosphorylated by the CDK2‐CycE complex, while the CDK2‐CycA complex has minimal effect (Figure 3). This suggests that WRN is highly phosphorylated during S phase when HR is dominant.

It has been reported that phosphorylation of WRN on serine residue 1133 by CDK1 plays a role in DNA2‐dependent end resection at replication‐related DSBs to promote HR (Palermo et al., 2016). In agreement with these findings, we also observed that CDK1‐CycA/B phosphorylated WRN, although to a lesser extent than CDK2‐CycE combination (Figure 3a). CDK1 acts in conjunction with CycA or CycB to finish S phase and drive cell cycle progression into mitosis and thus, its activity peaks in early M phase (Parry et al., 2003; Vazquez‐Novelle et al., 2014). Taken together, it is likely that a combination of both mechanisms regulates the phosphorylation of WRN. In early S phase, where the CDK2‐CycE complex is active, CDK2 is likely responsible for WRN phosphorylation. During late S to early G2 phase, where CDK1 becomes active, CDK1 phosphorylates WRN to drive DSB repair toward HR. Although the CDK2‐CycE starts to activate at late G1 phase, it is likely that the effect of WRN phosphorylation on HR would be minimal at this point due to the lack of sister chromatid.

Amino acid substitutions at S426 or S1133 reveal subtle differences with respect to DSB repair control. In the cellular repair assays, at both positions, the non‐phosphorylatable A substitution resulted in increased NHEJ and decreased HR (Figure 6d and e, Palermo et al., 2016). For S1133D WRN expressing cells, HR was twofold increased and NHEJ was unchanged. HR and NHEJ were like WT in cells expressing S426D. Both our group and Palermo et. al. showed that these substitutions did not alter the helicase or exonuclease activities of WRN. WRN interacts with many proteins and these amino acids do appear to regulate these interactions. S426A/D alters WRNs interaction with RPA (Figure 6c) and S426D showed increased accumulation to the sites of laser‐induced DSBs (Figure 6a,b). While S1133 alters WRN:DNA2 long‐range resection and, in MRE11 KD cells, S1133 mutants showed reduced recruitment to replication‐induced DSBs (Palermo et al., 2016). Thus, the non‐phosphorylatable WRN mutants promote NHEJ and both positions modulate protein:protein interactions to alter recruitment dynamics.

In our experiments, CDK2 inhibition not only altered WRN’s recruitment dynamics to the DSB, but also changed WRN’s localization in the pre‐laser images (Figure 1a and d). Endogenous WRN localizes to the nucleoli in unstressed cells (Marciniak et al., 1998; Partridge et al., 2003). In contrast with all the other agents, CDK2 inhibition changed WRN’s localization such that it was re‐distributed out of the nucleoli and into the nucleoplasma into puncta. The nature of this CDK2i‐dependent change could simply reflect WRN’s distribution as a function of the cell cycle (Lan et al., 2005). But it might also reflect additional post‐translational modifications as it is reported that the acetylation levels also regulate WRN’s intracellular localization by p300 (Blander et al., 2002) or SIRT1 (Li et al., 2008). Both of these proteins are regulated by CDK2 (Morris et al., 2000; Sasaki et al., 2008). Thus, there may be several interconnected complex regulatory networks modulating WRN’s recruitment and accumulation kinetics by CDK2 pre‐ and post‐laser treatment. We observed that the phosphomimetic mutant of WRN showed increased binding affinity to RPA (Figure 6c). As DNA end resection is a rate‐limiting factor in DSB pathway choice (Symington & Gautier, 2011) and RPA binds and stabilizes single‐stranded DNA formed by end resection for HR (Ruff et al., 2016), it is most likely that the CDK2‐mediated S426 phosphorylation of WRN increases the binding affinity to RPA, generating longer single‐stranded DNA for optimal recombination reactions.

It has also been shown that WRN suppresses initial resection by inhibiting MRE11 and CtIP recruitment to DSBs in G1 phase for efficient NHEJ (Shamanna et al., 2016). According to our data, S426 phosphorylation status does not affect WRN’s helicase activity (Figure 5a). Also, the phosphorylation state of S426 does not seem to affect WRN’s interaction affinity with KU70/80 (Figure 6c) or WRN’s exonuclease activity as a single protein or in coordination with KU70/80 (Figure 5b), suggesting that CDK2‐mediated S426 phosphorylation does not play a role in the initial short‐range resection process. Instead, S426 phosphorylation more likely regulates a later stage of HR. Once DNA end resection by WRN and RPA is finished, RPA is displaced with RAD51 by BRCA2 (Bhat & Cortez, 2018). Loss of WRN exonuclease activity further stimulates engagement of RAD51 (Aiello et al., 2019), then RAD51 subsequently facilitates strand invasion for HR. With WRN’s strand annealing activity affected by S426 phosphorylation (Figure 5c), it is likely that S426 phosphorylation stimulates strand annealing during RAD51 mediated D‐loop formation. Indeed, RAD51 is a known interaction partner of WRN (Otterlei et al., 2006) and WRN’s strand pairing activity becomes more effective than its helicase activity when the DNA length becomes longer than 50–70 bp (Machwe et al., 2005), such as during HR when long‐range resection occurs. Overall, our data support the importance of S426 in DSB pathway choice and we have shown that the phosphorylation defective WRN mutant has higher NHEJ and lower HR efficiency (Figure 6d and e).

Based on the above data, we present a model for the role of WRN in DSB repair pathway choice that is modulated by CDK2‐mediated phosphorylation (Figure 6f). If DSBs occur in the G1 phase of the cell cycle, WRN inhibits the recruitment of MRE11 and CtIP to the DSB to promote NHEJ. When cells progress into S phase of the cell cycle, CDK2 becomes active via CycE, and phosphorylates WRN on S426. S426 phosphorylated WRN has increased affinity for RPA, thereby promoting end resection and stabilization of the resected single‐stranded DNA. Also, S426 phosphorylation enhances WRN’s strand annealing activity to promote D‐loop formation for efficient HR. As such, our study shows that phosphorylation of WRN by CDK2 is key in regulating WRN’s ability to mediate the choice between DNA repair pathways.

## 4 MATERIALS AND METHODS

### 4.1 Cell culture and DNA transfections

U2OS and HEK293T cell lines were purchased from ATCC. U2OS‐based EJ5 and DR‐GFP cell lines were gifts from Dr. Jeremy Stark (City of Hope, Duarte, CA, USA) and Dr. Xiaofan Wang (Duke University, Durham, NC, USA). All cell lines were cultured in Dulbecco's modified Eagle's medium (Life Technologies, Carlsbad, CA, USA) containing 10% fetal bovine serum (FBS) in an atmosphere of 5% CO_2_ at 37°C. For real‐time WRN recruitment studies, 1 × 10^6^ cells were transfected with 1 µg of pcDNA3.1‐mCherry‐WRN plasmid using Amaxa Cell Line Nucleofector Kit V (Lonza, Basel, Switzerland) by following the company's transfection protocol. For inhibiting the activities of DNA‐PKcs, ATM, ATR, CDK1, and CDK2, the cells were, respectively, treated with NU7026 (Tocris, Bristol, United Kingdom), KU55933 (Tocris), VE821 (Tocris), RO3306 (Tocris), or CDK2 inhibitor 2‐III (EMD Millipore, Burlington, MA, USA) for 2 to 4 h, as stated in the legend. To achieve ectopic expression of 3×FLAG‐WRN, 1 × 10^7^ 293T cells were transfected with 5 µg of pcDNA3.1 carrying 3×FLAG or 3×FLAG‐WRN using JetPrime transfection reagent (Polyplus Transfections, New York, NY, USA).

### 4.2 Microirradiation and microscopy

DSBs were generated in 0.25 × 3 µM tracks using a Stanford Research Systems (SRS) NL100 nitrogen MicroPoint System (Andor Technology, Belfast, United Kingdom) equipped to a Nikon Eclipse TE2000 spinning disk confocal microscope (Nikon Instruments, Melville, NY, USA). The microscope was supported with temperature and CO_2_‐regulated incubation chamber, and the DSBs were induced with 435 nm laser regulated through Volocity software 6.3 (PerkinElmer, Waltham, MA, USA). Following the laser‐induced DSBs, mCherrry‐WRN recruitment was recorded at 5 to 15 sec intervals for 5 min with a CCD camera (Hamamatsu, Hamamatsu City, Shizuoka, Japan). The fluorescence intensity of the damaged area was measured with Volocity imaging software and normalized to that of a control area. The results are presented as mean ± SEM, and *p*‐values were measured with Student's *t* test.

### 4.3 In vitro phosphorylation, immunoblotting, and mass spectrometry

Generation of recombinant baculoviruses, protein expression in insect cells, and purification was carried out as described previously (Tadokoro et al., 2012). The previously described plasmid 6×His‐WRN‐FLAG/pFastBac1‐InteinCBDAla was modified using site‐directed mutagenesis (Tadokoro et al., 2012). The primer pairs indicated in Supplementary Table S1 were utilized to substitute serine 426 with alanine or aspartic acid, respectively. The resulting nucleotide sequences were verified by direct sequencing. For dephosphorylation of WRN, 2 µg of purified 3×FLAG‐WRN was treated with λ phosphatase (NEB, Ipswich, MA, USA) in PMP buffer (NEB) and MnCl_2_ for 20 min at 30℃. Dephosphorylated WRN was resuspended in 1 ml immunoprecipitation buffer (25 mM HEPES, 250 nm NaCl, 0.25% NP40, 5% glycerol, and 1×protease inhibitor) and incubated with 20 µl FLAG M2 beads overnight at 4℃. Beads were centrifuged at 1000×g for 5 min at 4°C and washed twice with wash buffer A (50 mM HEPES, 500 mM NaCl, and 0.5% NP40) and once with wash buffer B (50 mM HEPES, 50 mM KCl). Beads were resuspended in *in vitro* phosphorylation buffer containing 25 mM HEPES pH 7.4, 5 mM MgCl_2_, 5 mM sodium pyrophosphate, 2 mM ATP, 2 mM DTT, and phosphatase inhibitor. WRN was phosphorylated by incubating ¼ of the immunoprecipitated WRN with 100 ng of CDK1‐CycA (Thermo Fisher Scientific, Waltham, MA, USA), CDK1‐CycB (Thermo Fisher scientific), CDK2‐CycA (EMD millipore), or CDK2‐CycE (EMD millipore). Reactions were performed for 30 min at 30°C in *in vitro* phosphorylation buffer and terminated by adding 2×Laemmli buffer and processed for immunoblotting. For FLAG‐WRN pull‐down experiments, U2OS cells were 10 Gy γ‐irradiated, harvested 30 min post‐irradiation, and lysed with buffer A containing 1×phosphatase inhibitor cocktail and 20 U mL^−1^ Benzonase (EMD millipore), in the presence of 1 mM MgCl_2_. After removing cell debris by centrifuging, the lysates were incubated with FLAG‐WRN proteins and incubated 4°C overnight. Samples were incubated with magnetic FLAG M2 beads (Millipore‐Sigma, St. Louis, MO, USA) 1 h at 4°C. Beads were washed five times with buffer A and one time with TE pH 8.0, then eluted with TE pH 8.0 containing 250 µg mL^−1^ 3×FLAG peptides. Proteins were resolved in 4 to 15% Mini‐PROTEAN TGX gels (BioRad, Hercules, CA, USA), electroblotted to nitrocellulose membrane, and visualized using antibodies against anti‐WRN (in house), anti‐phospho‐CDK substrate (Cell Signaling, 9477), anti‐CDK1 (Abcam, ab133327), anti‐CDK2 (Abcam, ab32147), anti‐CycA2 (Abcam, ab38), anti‐CycE (Abcam, ab133266), anti‐DNA‐PKcs (BD, 610804), anti‐KU70 (ThermoFisher, PA5‐27538), and anti‐RPA (Calbiochem, NA18). Anti‐mouse/rabbit IgG (HRP‐linked) from Cell Signaling (Danvers, MA, USA) secondary antibodies were used with a dilution of 1:2000. For mass spectrometry, the 3×FLAG‐WRN phosphorylated by CDK2‐CycE complex was resolved on SDS‐PAGE, stained with SimplyBlue™ *SafeStain* (Invitrogen, Carlsbad, CA, USA) and WRN‐specific bands were excised and submitted to Harvard Taplin Mass Spectrometry Facility (Boston, MA, USA). Phosphorylated serine/threonine blot intensities were quantified with ImageJ (Schneider et al., 2012) by normalizing with WRN and basal phosphorylation without CDKs. For quantifying phosphorylated serine blot intensity after dephosphorylation, Western blot image was quantified by normalizing with WRN. The results are presented as mean ± SD with p‐values determined by Student's *t* test.

### 4.4 DSB repair assays

The HR repair and NHEJ assays were performed in DR‐GFP U2OS cells and EJ5 U2OS cells, respectively. mCherry‐WRN, along with the plasmids expressing I‐SceI endonuclease were transfected into 1 × 10^6^ DR‐GFP U2OS cells or EJ5 U2OS cells with JetPrime transfection reagent (Polyplus Transfections) according to the manufacturer's protocols. 48 h after the transfection, the cells were submitted for flow cytometry with LSR Fortessa Flow Cytometer (BD bioscience, San Jose, CA, USA). The results are presented as mean ± SD from four independent experiments with p‐values determined by Student's *t* test.

### 4.5 Oligonucleotide substrates for *in vitro* biochemical assays

Oligonucleotides listed in Supplementary Table S1 were ordered from Eurofins Genomics (Louisville, KY, USA). T30‐D50PT‐Top, Exo‐32, and C80 were 5’ radiolabeled with [γ‐32P] ATP (3000 Ci/mmol, PerkinElmer) and T4 polynucleotide kinase (New England Biolabs, Ipswich, MA, USA). Labeled oligos were annealed with corresponding primers (1:1.2 ratio) in 40 mM Tris‐HCl pH 8.0 and 50 mM NaCl by heating at 85 ℃ for 2 minutes and cooling to 20 ℃ at a rate of 1℃/min. Unincorporated [γ‐32P] ATP was removed using MicroSpin G‐25 columns (GE Healthcare, Chicago, IL, USA).

### 4.6 Helicase assays

T30‐D50PT‐Top/ T30‐D50PT‐Bottom forked DNA duplex substrate (1 nM) was incubated with indicated amounts of WRN and RPA proteins in 20 µl of reaction buffer (40 mM Tris‐HCl, pH 8.0, 2 mM ATP, 4 mM MgCl_2_, 5 mM DTT, 100 μg/ml BSA, 10% glycerol) for 30 minutes at 37℃. Reactions were stopped by addition of 10 µl of stop buffer (30 mM EDTA pH 8.0, 30 mM Tris‐HCl pH 8.0, 30% glycerol, 0.9% SDS, 0.05% bromophenol blue). Products were separated by electrophoresis on 8% native polyacrylamide gels and exposed to a phosphorimager plate. Images were acquired on Typhoon FLA 9500 (Cytiva, Marlborough, MA, USA) and analyzed using ImageQuant TL software (GE Healthcare).

### 4.7 Strand annealing assays

Radiolabeled single‐stranded C80 oligonucleotide (1 nM) and unlabeled G80 oligonucleotide (1.2 nM) were incubated with indicated WRN proteins in 20 µl of reaction buffer (20 mM Tris‐HCl, pH 8.0, 1 mM DTT, 40 μg/ml BSA) for 30 minutes at 37℃. Reactions were stopped by addition of 10 µl of stop buffer (30 mM EDTA, pH 8.0, 30 mM Tris‐HCl pH 8.0, 30% glycerol, 0.9% SDS, 0.05% bromophenol blue). Products were separated by electrophoresis on 8% native polyacrylamide gels and exposed to a phosphorimager plate. Images were acquired on Typhoon FLA 9500 (Cytiva) and analyzed using ImageQuant TL software (GE Healthcare).

### 4.8 Exonuclease assays

Exo‐32/Exo‐43 5’‐overhang substrate (0.5 nM) was incubated with indicated amounts of WRN and Ku proteins in 20 µl of reaction buffer (40 mM Tris‐HCl, pH 8.0, 2 mM ATP, 4 mM MgCl_2_, 5 mM DTT, 100 μg/ml BSA, 10% glycerol) for 30 minutes at 37℃. Reactions were stopped by addition of 10 µl of stop buffer (98% formamide, 10 mM EDTA, pH 8.0, 0.05% bromophenol blue, 0.05% orange G) and heated for 5 minutes at 95℃. Products were separated by electrophoresis on 15% denaturing polyacrylamide gels and exposed to a phosphorimager plate. Images were acquired on Typhoon FLA 9500 (Cytiva) and analyzed using ImageQuant TL software (GE Healthcare).

## CONFLICT OF INTEREST

The authors declare no competing interests.

## AUTHOR CONTRIBUTIONS

J.H.L. and R.A.S. designed experiments. J.H.L. and R.A.S. performed most of the experiments and analyzed data. J.H.L. wrote the manuscript. T.K. performed biochemical assays. N.B.F. performed laser‐induced mutant WRN and RPA recruitment experiments. E.W.K. performed coimmunoprecipitation and helped editing the manuscript. T.K. and L.C.S. purified FLAG‐WRN proteins. D.L.C. contributed to discussions and writing manuscript. V.A.B. supported study design, data analysis and writing, and acquired financial support.

## Supporting information

Fig S1‐S2

Table S1

Table S2

## Data Availability

Data sharing is not applicable to this article as no datasets were generated or analyzed during the current study.
