# Supplementary material for: CDK2 phosphorylation of Werner protein (WRN) contributes to WRN’s DNA double‐strand break repair pathway choice
Source: Aging Cell. 2021 Oct 6;20(11):e13484. doi: 10.1111/acel.13484 (PMC8590104; doi:10.1111/acel.13484)
Supplement: Supplementary file 1 — Fig S1‐S2 [file ACEL-20-e13484-s002.pdf]

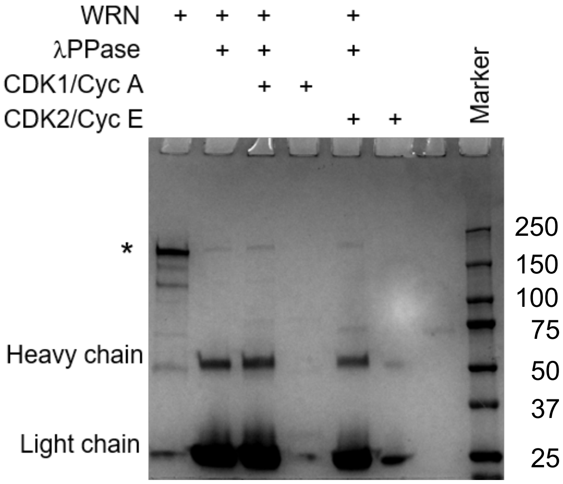

Supplementary figure 1. Identification of CDK2 phosphorylation site on WRN. Purified WRN proteins were *in vitro* phosphorylated with by CDK1-CycA or CDK2-CycE complex and subsequently loaded and resolved on SDS-PAGE and stained with Coomassie brilliant blue. WRN protein bands were analyzed with mass spectrometric analysis. Asterisks indicate WRN proteins.

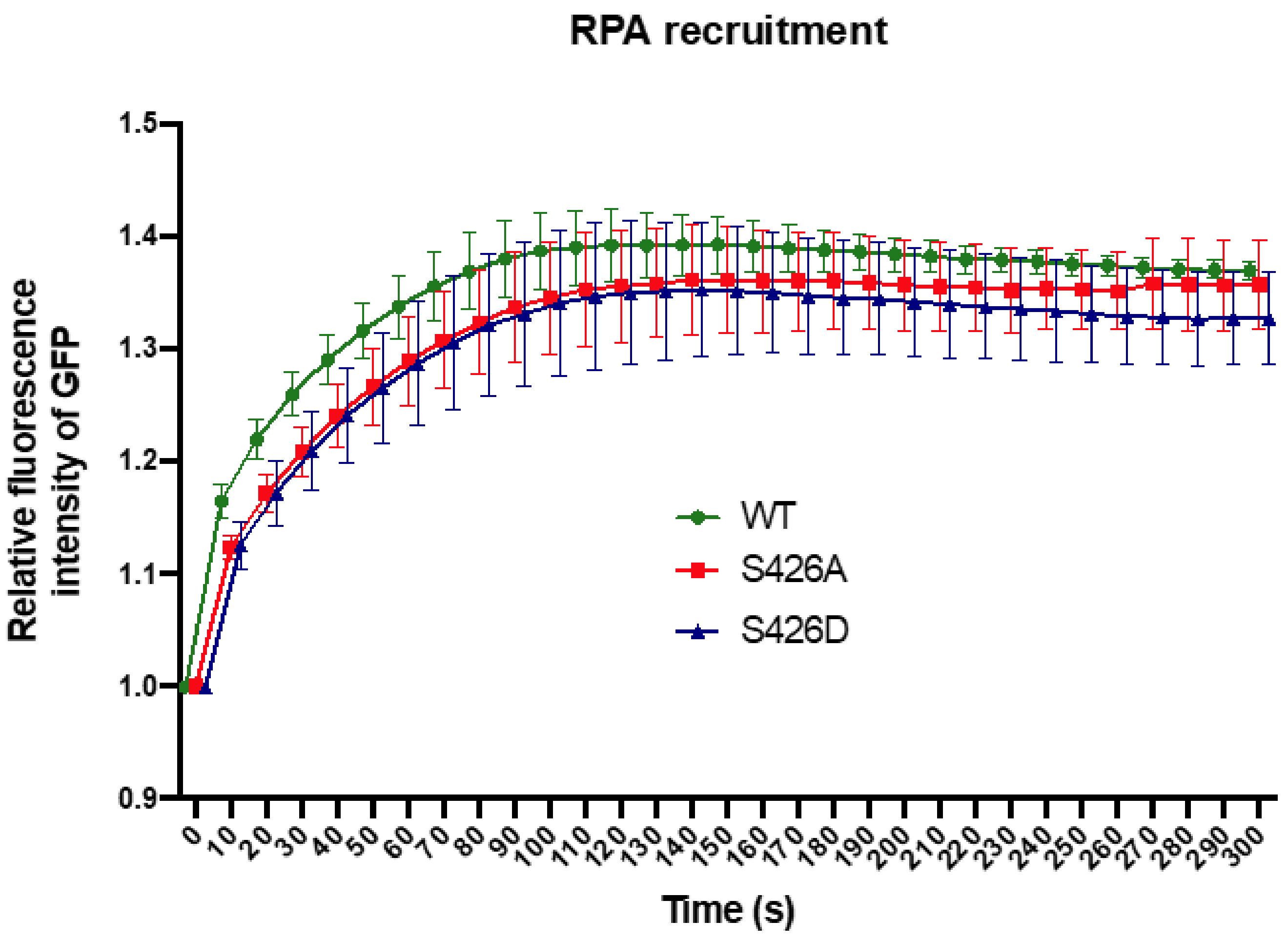

Supplementary figure 2. RPA recruitment to the DSB is not affected by WRN mutation. Recruitment of GFP-RPA to laser tracks. U2OS cells expressing WT/S426A/D-WRN and GFP-RPA were micro-irradiated with 435 nm laser to induce DSBs. Images were captured at 10 sec intervals for 5min.
