# Supplementary material for: CDK2 phosphorylation of Werner protein (WRN) contributes to WRN’s DNA double‐strand break repair pathway choice
Source: Aging Cell. 2021 Oct 6;20(11):e13484. doi: 10.1111/acel.13484 (PMC8590104; doi:10.1111/acel.13484)
Supplement: Supplementary file 2 — Table S1 [file ACEL-20-e13484-s001.docx]

**Table 1**

Oligonucleotide sequences

| Name | Sequence (5’ > 3’) |
| --- | --- |
| hWRN-S426A-F  hWRN-S426A-R  hWRN-S426D-F  hWRN-S426D-R  T30-D50PT-Top  T30-D50PT-Bottom  C80  G80  Exo-32  Exo-43 | taaatctactgagcatttagctcccaatgataat  ctaaatgctcagtagatttataagcaatatcact  aaatctactgagcatttagatcccaatgataatg  tctaaatgctcagtagatttataagcaatatcac  TTTTTTTTTTTTTTTTTTTTTTTTTTTTTTGGCAAACATGTCCTAGCAAGGCACTGGTAGAATTCGGCAGCGTGCTTCTC  GAGAAGCACGCTGCCGAATTCTACCAGTGCCTTGCTAGGACATGTTTGCCTTTTTTTTTTTTTTTTTTTTTTTTTTTTTT  CCCCCCCCCCCCCCCCCCCCCCCCCCCCCCCCCCCCCCCCCCCCCCCCCCCCCCCCCCCCCCCCCCCCCCCCCCCCCCCC  GGGGGGGGGGGGGGGGGGGGGGGGGGGGGGGGGGGGGGGGGGGGGGGGGGGGGGGGGGGGGGGGGGGGGGGGGGGGGGGG  TGACGTGACGACGATCAGGGTACGTTCAGCAG  AGTGCAGACTGCTGCTGAACGTACCCTGATCGTCGTCACGTCA |
